# Variation in the Use of Active Surveillance for Low-Risk Prostate Cancer Across US Census Regions

**DOI:** 10.3389/fonc.2021.644885

**Published:** 2021-05-19

**Authors:** Bashir Al Hussein Al Awamlh, Neal Patel, Xiaoyue Ma, Adam Calaway, Lee Ponsky, Jim C. Hu, Jonathan E. Shoag

**Affiliations:** ^1^Department of Urology, Weill Cornell Medicine, New York Presbyterian Hospital, New York, NY, United States; ^2^Division of Biostatistics and Epidemiology, Department of Healthcare Policy and Research, Weill Cornell Medicine, New York, NY, United States; ^3^Urology Institute, University Hospitals Cleveland Medical Center, Cleveland, OH, United States; ^4^Department of Urology, Case Western Reserve University School of Medicine, Cleveland, OH, United States

**Keywords:** low-risk prostate cancer, active surveillance, geographic variation, watchful waiting, radical prostatectomy, radiation therapy

## Abstract

Substantial geographic variation in healthcare practices exist. Active surveillance (AS) has emerged as a critical tool in the management of men with low-risk prostate cancer. Whether there have been regional differences in adoption is largely unknown. The SEER “Prostate with Watchful Waiting Database” was used to identify patients diagnosed with localized low-risk prostate cancer and managed with AS across US census regions between 2010 and 2016. Multivariable logistic regression models were used to determine the impact of region on undergoing AS and factors associated with AS use within each US census region. Between 2010 and 2016, the proportion of men managed with AS increased from 20.8% to 55.9% in the West, 11.5% to 50.0% in Northeast, 9.9% to 43.4% in the South and 15.1% to 56.2% in Midwest (*p* < 0.0001). On multivariable analysis, as compared to the West, men in all regions were less likely to undergo AS (*p* < 0.001). Black men in the West (OR 1.36, 95%CI 1.25–1.49) and Midwest (OR 1.62, 95%CI 1.35–1.95) were more likely to undergo AS, but less likely in Northeast (OR 0.80, 95%CI 0.69–0.92). Men with higher socioeconomic status (SES) were more likely to undergo AS in the West (OR 1.47, 95%CI 1.39–1.55), Northeast (OR 1.57, 95%CI 1.36–1.81), and South (OR 1.24, 95%CI 1.13–1.37) but not in the Midwest (OR 0.85, 95%CI 0.73–0.98). We found striking regional differences in the uptake of AS according to race and SES. Geography must be taken into consideration when assessing barriers to AS use.

## Introduction

Active surveillance (AS) has been widely adopted for low-risk prostate cancer in an effort to mitigate the harms of overtreatment (1). Recent data has demonstrated an increase in AS use in the US from 15 to 42% between 2010 and 2015 (2). While this increase is encouraging, the US lags behind other countries which have adopted AS more robustly such as Sweden where 74% of men with low-risk prostate cancer were managed in this fashion during the same timeframe (3).

Geographic variation in healthcare delivery has been implicated as a source of idiosyncratic variation in healthcare utilization, outcomes, and spending for over 50 years (4, 5). Prostate cancer care has been shown to vary dramatically at the regional, county and facility levels, and even within the same practice (6–8).

Recently, we demonstrated that when accounting for geographic variation, Black men were more likely than white men to undergo AS (9). These data highlight the importance of determining how men with low-risk prostate cancer are managed differently according to region. Here, we sought to elucidate regional differences, and contributors to AS use in men with low-risk prostate cancer across census regions in the US using nationally representative data.

## Methods

The SEER “Prostate with Watchful Waiting Database” was used to identify patients diagnosed with localized low-risk (clinical T1c-T2a, Gleason score 3+3 and PSA < 10 ng/mL) prostate cancer between 2010 and 2016. SEER collects and publishes cancer incidence, prevalence, and survival data from population-based cancer registries, that are nationally representative, covering approximately one third of the U.S. population. The “Prostate with Watchful Waiting Database,” is a specialized database that contains active surveillance (or watchful waiting) information collected by SEER for prostate cancer cases diagnosed from 2010 to 2016 (10). Men with missing clinical data, including those whose initial management was unknown, and those that were not treated by their physicians for reasons such as the presence of comorbidities were excluded from analysis. This study was approved the Institutional Review Board at Weill Cornell Medicine and the SEER custom data group.

Subjects were categorized into one of four US census regions (Northeast, West, South, and Midwest) according to the location of the SEER registry reported. Clinical demographic factors were compared among men undergoing AS and definitive treatment (radical prostatectomy or radiation therapy). Clinical variables included age at the time of diagnosis, prostate specific antigen (PSA) level, and total and positive numbers of prostate biopsy cores. Demographic variables included insurance status (insured, Medicaid, uninsured, unknown), race (White, Black, and other/unknown) and socioeconomic status (SES) which was measured using the Yost index (composite score based on census tract-level median household income, median house value, median rent, percent below 150% of poverty line, education Index, percent working class, and percent unemployed) (11).

The main outcomes of the study were to determine how men were managed across different regions in the US between 2010 and 2016, and how clinical and demographic factors were associated with undergoing AS within each region. Clinical and demographic factors were compared using the chi-square test, *t-*test or Mann-Whitney test as indicated. Temporal trends were compared using the Cochran-Armitage trend test. Multivariable logistic regression was used to determine the impact of region on undergoing AS and in a separate analysis the cohort was stratified according to age. Multivariable models were also used to identify factors associated with AS use within each US census region. Statistical analyses were performed using R version 3.4.4 and SAS Version 9.4. All *p-*values are two-sided with statistical significance evaluated at α = 0.05.

## Results

Characteristics of men diagnosed with low-risk prostate cancer are shown in Table 1. Black men constituted 27.2% of men diagnosed with low-risk prostate cancer in the South, 13.0% in the Northeast, 17.5% in the Midwest and 9.1% in the West (*p* < 0.001). Among men diagnosed with low-risk prostate cancer, a higher proportion were of higher SES in the Northeast at 87.0% and the West at 56.6% as compared to the Midwest at 34.4% in and the South at 25.2% (*p* < 0.001). Between 2010 and 2016, the majority of men with low-risk prostate cancer were managed with radical prostatectomy in the West and Midwest at 38.8 and 42.2%, respectively. Whereas, in the Northeast the majority of men were managed with radiation therapy at 37.6% and in the South men were managed almost equally with radiation therapy at 38.0% and radical prostatectomy at 38.3% (*p* < 0.001).

**Table 1:** Characteristics of men with low-risk prostate cancer in each US census region between 2010 and 2016.

	**West (*n* = 29,320)**	**Northeast (*n* = 12,428)**	**South (*n* = 14,399)**	**Midwest (*n* = 4,633)**	***P*-value**
Median age, years (IQR)	63.0 (57.0–68.0)	63.0 (57.0–68.0)	63.0 (57.0–68.0)	62.0 (57.0–68.0)	<0.001
Age, n (%)					<0.001
<60 years	10,067 (34.3%)	4,311 (34.7%)	5,062 (35.2%)	1,730 (37.3%)	
60–69 years	14,373 (49.0%)	5,706 (45.9%)	6,762 (47.0%)	2,110 (45.5%)	
≥70 years	4,880 (16.6%)	2,411 (19.4%)	2,575 (17.9%)	793 (17.1%)	
Year, n (%)					<0.001
2010–2012	16,095 (54.9%)	6,467 (52.0%)	7,388 (51.3%)	2,582 (55.7%)	
2013–2015	10,269 (35.0%)	4,493 (36.2%)	5,434 (37.3%)	1,576 (34.0%)	
2016	2,956 (10.1%)	1,468 (11.8%)	1,577 (11.0%)	475 (10.3%)	
Race, n (%)					<0.001
White	23,631 (80.6%)	10,192 (82.0%)	10,343 (71.8%)	3,743 (80.8%)	
Black	2,659 (9.1%)	1,620 (13.0%)	3,910 (27.2%)	810 (17.5%)	
Other/Unknown	3,030 (10.3%)	616 (5.0%)	146 (1.0%)	80 (1.7%)	
Median PSA, ng/mL (IQR)	5.5 (4.5–7.0)	5.0 (4.1–6.3)	5.2 (4.3–6.6)	5.2 (4.2–6.6)	<0.001
Number of positive cores					<0.001
2 or less positive cores	13,927 (47.5%)	5,793 (46.6%)	6,698 (46.5%)	2,521 (54.4%)	
3 or more positive cores	9,952 (33.9%)	3,399 (27.4%)	4,653 (32.3%)	1,424 (30.7%)	
Unknown	5,441 (18.6%)	3,236 (26.0%)	3,048 (21.2%)	688 (14.9%)	
Socioeconomic status, n (%)					<0.001
High SES	16,597 (56.6%)	10,814 (87.0%)	3,628 (25.2%)	1,593 (34.4%)	
Low SES	12,720 (43.4%)	1,613 (13.0%)	10,771 (74.8%)	3,040 (65.6%)	
Insurance, n (%)					<0.001
Insured	27,323 (93.2%)	9,892 (79.6%)	13,159 (91.4%)	4,195 (90.6%)	
Medicaid	1,027 (3.5%)	305 (2.5%)	521 (3.6%)	151 (3.3%)	
Uninsured	174 (0.60%)	181 (1.5%)	187 (1.3%)	40 (0.9%)	
Unknown	796 (2.7%)	2,050 (16.5%)	532 (3.7%)	247 (5.3%)	
Treatment, n (%)					<0.001
Active Surveillance	10,693 (36.5%)	3,362 (27.1%)	3,407 (23.6%)	1,377 (29.7%)	
Radical prostatectomy	11,385 (38.8%)	4,393 (35.4%)	5,519 (38.3%)	1,962 (42.4%)	
Radiation therapy	7,242 (24.7%)	4,673 (37.6%)	5,473 (38.0%)	1,294 (27.9%)	

*IQR, Interquartile range*.

*Percentages may not add up to 100% due to rounding*.

**Figure 1:**
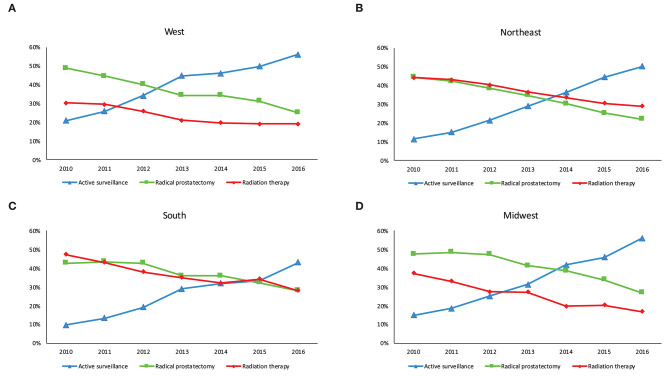
Management of men with low-risk prostate cancer in each US census region between 2010 and 2016. **(A)** West **(B)** Northeast **(C)** South and **(D)** Midwest.

The proportion of men managed with AS increased from 20.8 to 55.9% in the West, 11.5–50.0% in Northeast, 9.9–43.4% in the South and 15.1–56.2% in Midwest (*p* < 0.001 trend test, Figure 1). The differences in characteristics in men undergoing AS by region are shown in Table 2 and by race in Supplementary Table 1.

**Table 2:** Characteristics of men managed with active surveillance in each US census region between 2010 and 2016.

	**West (*n* = 10,693)**	**Northeast (*n* = 3,362)**	**South (*n* = 3,407)**	**Midwest (*n* = 1,377)**	***P*-value**
Median age, years (IQR)	64 (59.0–68.0)	65 (59.0–69.0)	65.0 (60.0–70.0)	65.0 (59.0–69.0)	<0.001
Age, n (%)					
<60 years	3,060 (28.6%)	917 (27.3%)	819 (24.0%)	366 (26.6%)	
60–69 years	5,522 (51.6%)	1,618 (48.1%)	1,653 (48.5%)	675 (49.0%)	
≥70 years	2,111 (19.7%)	827 (24.6%)	935 (27.4%)	336 (24.4%)	
Year, n (%)					<0.001
2010–2012	4,253 (39.8%)	1,002 (29.8%)	1,022 (30.0%)	491 (35.7%)	
2013–2015	4,787 (44.8%)	1,626 (48.4%)	1,700 (48.9%)	619 (45.0%)	
2016	1,653 (15.5%)	734 (21.8%)	685 (20.1%)	267 (19.4%)	
Race, n (%)					<0.001
White	8,435 (78.9%)	2,814 (83.7%)	2,463 (72.3%)	1,061 (77.1%)	
Black	1,028 (9.6%)	352 (10.5%)	890 (26.1%)	285 (20.7%)	
Other/Unknown	1,028 (11.5%)	196 (5.8%)	54 (1.6%)	31 (2.3%)	
Median PSA, ng/mL (IQR)	5.60 (4.6–7.0)	5.1 (4.2–6.5)	5.4 (4.4–6.7)	5.3 (4.4–6.8)	<0.001
Number of positive cores, n (%)					<0.001
2 or less positive cores	6,936 (64.9%)	2,236 (66.5%)	2,262 (66.4%)	994 (72.3%)	
3 or more positive cores	2,436 (22.8%)	580 (17.3%)	624 (18.3%)	258 (18.7%)	
Unknown	1,321 (12.4%)	546 (16.2%)	521 (15.3%)	125 (9.1%)	
Socioeconomic status, n (%)					<0.001
High SES	6,664 (62.3%)	3,042 (90.5%)	957 (28.1%)	436 (31.7%)	
Low SES	4,029 (37.7%)	319 (9.5%)	2,450 (71.9%)	941 (68.3%)	
Insurance, n (%)					<0.001
Insured	9,878 (92.4%)	2,744 (81.6%)	3,098 (90.9%)	1,170 (85.0%)	
Medicaid	320 (3.0%)	107 (3.2%)	91 (2.7%)	53 (3.9%)	
Uninsured	60 (0.6%)	97 (2.9%)	48 (1.4%)	12 (0.9%)	
Unknown	435 (4.1%)	414 (12.3%)	170 (5.0%)	142 (10.3%)	

*IQR, Interquartile range*.

*Percentages may not add up 100% due to rounding*.

On multivariable regression including US census regions, men in the South [adjusted odds ratio [aOR] 0.51, 95% confidence interval [CI] 0.49–0.54], Northeast (aOR 0.50, 95%CI 0.48–0.53), and Midwest (aOR 0.71, 95% CI 0.66–0.76) were less likely to undergo AS as compared to men in the West. Furthermore, on multivariable analysis that is stratified by age group (Supplementary Table 2), similar associations between clinical and sociodemographic factors, and the receipt of AS were noted across different ages. In region specific models, Black men in the West (aOR 1.36, 95%CI 1.25–1.49) and Midwest (aOR 1.62, 95%CI 1.35–1.95) were more likely to undergo AS than White men, but were less likely to undergo AS in the Northeast (aOR 0.80, 95%CI 0.69–0.92) as compared to White men (Table 3). Men with higher SES were more likely to undergo AS in the West (aOR 1.47, 95%CI 1.39–1.55), Northeast (aOR 1.57, 95%CI 1.36–1.81), and South (aOR 1.24, 95%CI 1.13–1.37) but not in the Midwest (aOR 0.85, 95%CI 0.73–0.98). Multivariable models including an interaction term for race and SES (Supplementary Table 3) showed significant interactions between race and SES in all regions except the Midwest.

**Table 3:** Multivariable logistic regression analysis assessing receipt of active surveillance.

	**West**	**Northeast**	**South**	**Midwest**
Region^*^	Reference	0.50 (0.48–0.53)	0.51 (0.49–0.54)	0.71 (0.66–0.76)
Age (per one year)	1.04 (1.03–1.04)	1.04 (1.03–1.05)	1.06 (1.06–1.07)	1.06 (1.05–1.07)
Year				
2010–2012	Reference	Reference	Reference	Reference
2013–2015	2.58 (2.44–2.73)	3.21 (2.91–3.53)	3.06 (2.79–3.35)	3.04 (2.61–3.54)
2016	3.79 (3.48–4.13)	5.90 (5.17–6.73)	5.25 (4.62–5.97)	6.19 (4.94–7.76)
Race				
White	Reference	Reference	Reference	Reference
Black	1.36 (1.25–1.49)	0.80 (0.69–0.92)	1.08 (0.98–1.19)	1.62 (1.35–1.95)
Other/Unknown	1.09 (1.00–1.19)	1.20 (0.99–1.45)	1.78 (1.22–2.60)	1.27 (0.77–2.11)
PSA (per 1 ng/mL)	0.98 (0.97–0.99)	1.01 (0.99–1.03)	0.97 (0.95–0.99)	1.00 (0.96–1.03)
Number of positive cores				
3 or more positive cores	Reference	Reference	Reference	Reference
2 or less positive cores	3.33 (3.14–3.53)	3.41 (3.05–3.81)	3.64 (3.28–4.04)	3.44 (2.90–4.07)
Unknown	1.20 (1.11–1.30)	1.50 (1.31–1.73)	1.67 (1.46–1.91)	1.35 (1.04–1.74)
Socioeconomic status				
Low SES	Reference	Reference	Reference	Reference
High SES	1.47 (1.39–1.55)	1.57 (1.36–1.81)	1.24 (1.13–1.37)	0.85 (0.73–0.98)
Insurance				
Insured	Reference	Reference	Reference	Reference
Medicaid	0.76 (0.66–0.88)	1.26 (0.97–1.64)	0.79 (0.61–1.01)	1.22 (0.84–1.79)
Uninsured	1.16 (0.83–1.63)	4.17 (3.01–5.78)	1.48 (1.03–2.12)	1.29 (0.62–2.72)
Unknown	1.67 (1.43–1.95)	0.59 (0.52–0.67)	1.26 (1.03–1.55)	3.18 (2.37–4.26)

* *separate multivariable logistic regression analysis assessing receipt of active surveillance in the overall cohort including region as a variable*.

## Discussion

Here, we demonstrate that by 2016, the West and Midwest had the highest rate of AS use among men with low-risk prostate cancer in the US at 56%. Whereas, the South was lagging behind other regions at 43%. We also found that clinical factors such as patient age, number of biopsies, and PSA level were associated with undergoing AS irrespective of US census region, while the contribution of demographic factors, such as SES, race, and patient insurance, to surveillance use varied across regions.

Recently, Loeb et al. (12) demonstrated in patients in an integrated health system, there are differences in the management of low-risk prostate cancer according to geography among US veterans. Similar to our data, the authors found that the use of AS was highest in the West and lowest in the Northeast. Factors explaining geographic variations in care are likely multifactorial and complex, and include availability of resources, treating physician specialty (urologist vs. radiation oncologist) (6), and individual patient and clinician preferences (7). Moreover, in a similar study that linked SEER AS data to county area data, the authors used different statistical analyses and also found that AS independently varied across different regions in the US (13). Our results underscore that regional variation in low-risk prostate cancer care may be driven by differential weighting of demographic factors across regions. The different associations observed among these factors in relation to the use of AS within each region are impressive, and highlight the need to understand and mitigate racial and SES barriers to AS within each region.

The observed geographic variation in the use of AS is impressive, but similar trends in the adoption of new treatment modalities have been previously seen in other malignancies. For instance, in the 1980's the use of breast-conserving surgery, which may serve as a model for studying the adoption new treatments, was shown to substantially vary in different regions in the US following its initial recommendation by experts (14). Similarly, by 2010 half of the colon resections performed in the US were laparoscopic, however, wide geographic variation in the use of laparoscopic colectomy for colon cancer persisted at that time ranging from 0 to 69% (15). Such trends in other cancer treatments also suggest that treatment location may considerably influence a patient's options for treatment approach and unequal access to new treatments is expected when a new treatment is first adopted.

Current AS guidelines do not recommend differential treatment of Black men, but suggest discussing the implications of potentially harboring higher-grade tumors when enrolling Black men into AS protocols (16). Here, we found that Black men undergo treatment differently in different regions of the US, independent of SES and other clinical factors. If a Black man is diagnosed with low-risk prostate cancer in the West or Midwest, he is more likely to be managed with AS. However, if he receives care in the Northeast, he is less likely to undergo AS than his White counterparts. This variation in care for Black men, compared to White men, in different regions maybe partly resultant from uncertainty in the management of Black men with low-risk cancer and the heterogeneity among Black men community in the US in different regions (17). These results further emphasize the importance of considering geography when assessing racial disparities in prostate cancer care (9).

Similar to previous studies, we also found that a higher SES is associated with undergoing AS (18). The difference in care according to SES is more pronounced in the Northeast and West. Interestingly, SES has no impact on the choice of treatment in the Midwest. These differences according to SES seen in other regions are perhaps related to access to academic or tertiary centers that are more likely to provide guideline concordant care (AS) (8). Alternatively, lower SES may be associated with the inability to follow up with the AS protocol, thus receiving upfront definitive treatment (19). Of note, while we observed significant differences based on insurance status, it is difficult to interpret these associations given that they occurred in men with unknown insurance.

This study has several limitations. First, it is a retrospective analysis subject to the limitations associated with data collection (20). Second, we could not determine the difference between AS or watchful waiting, and we were not able to deduce that based on frequency of testing with PSA or prostate biopsies after initial enrollment, as these data are not available. However, the median age of men on AS was 65, which is consistent with AS rather than watchful waiting given the life expectancy. Population-based studies have shown that only a small number of men (up to one third) on AS adhere to AS protocols, suggesting that differentiating AS from WW in a community setting beyond initial intent of treatment is challenging (21, 22). It is worth noting here that AS studies found Black men to be monitored less intensely than white men and were more likely to be lost to follow up (23, 24). We found that Black men on AS have significantly lower SES compared to others, therefore, it is plausible that the data overestimated Black men undergoing AS as opposed to watchful waiting. Third, we were not able to determine who undergoes definitive treatment after initially being managed with AS. Finally, and perhaps most importantly, unlike other studies that use data on smaller geographic areas that is more detailed (7), we were limited by the geographic information available to us and study with more granular geographic information may have benefits. This also precluded a more detailed analyses of regional factors (rather than patient factors) that contributed to such variability, such as treating facility, average regional distances traveled to receive care, or number of physicians or robots in the region. These limitations notwithstanding, these data are nationally representative and demonstrate substantial variations in the association of demographic factors in care across the US for low-risk prostate cancer. This study provides opportunities for policy makers and professional societies to study and better understand how to limit the unwarranted role of demographic factors in the use of AS and reduce variation in the guideline recommended care.

In conclusion, We found an increase in uptake of AS across all regional in the US between 2010 and 2016. Men in the West are more likely to managed with AS compared to other regions. Moreover, there are striking regional differences in the uptake of AS according to race and SES. Further study is needed to better understand these geographic variations in care in order to hone in efforts to eliminate the demographic barriers to AS.

## Data Availability Statement

The data analyzed in this study is subject to the following licenses/restrictions: The Surveillance, Epidemiology and End Results (SEER)-Medicare-linked database combines clinical information from population-based cancer registries with claims information from the Medicare program. The dataset has to be applied. Requests to access these datasets should be directed to https://healthcaredelivery.cancer.gov/seermedicare/contact.html.

## Ethics Statement

The studies involving human participants were reviewed and approved by Weill Conrell Medicine institutional review Board. Written informed consent for participation was not required for this study in accordance with the national legislation and the institutional requirements.

## Author Contributions

BA, NP, XM, and JS analyzed the data and drafted the manuscript. AC and LP helped interpreted the data. BA, JH, and JS designed the study. All authors contributed to the article and approved the submitted version.

## Conflict of Interest

The authors declare that the research was conducted in the absence of any commercial or financial relationships that could be construed as a potential conflict of interest.
