# Supplementary material for: Variation in the Use of Active Surveillance for Low-Risk Prostate Cancer Across US Census Regions
Source: Front Oncol. 2021 May 19;11:644885. doi: 10.3389/fonc.2021.644885 (PMC8170083; doi:10.3389/fonc.2021.644885)
Supplement: Supplementary file 1 [file Data_Sheet_1.PDF]

**Supplementary Table 1: Characteristics of men managed with active surveillance in US between 2010 and 2016 stratified by race.**

| | Black (n=2555) | Other/Unknown (n=1511) | White (n= 14773) | P value |
|---|---|---|---|---|
| Median age, years (IQR) | 62.0 (57.0-67.0) | 64 (59.0-69.0) | 65 (59.0-69.0) | <0.001 |
| Age, n (%) | | | | |
| < 60 years | 1237 (48.4%) | 768 (50.8%) | 7463 (50.5%) | |
| 60-69 years | 931 (36.4%) | 416 (27.5%) | 3815 (25.8%) | |
| ≥70 years | 387 (15.2%) | 327 (21.6%) | 3495 (23.7%) | |
| Year, n (%) | | | | 0.020 |
| 2010-2012 | 878 (34.4%) | 578 (38.3%) | 5312 (36.0%) | |
| 2013-2015 | 1174 (46.0%) | 680 (45.0%) | 6878 (46.6%) | |
| 2016 | 503 (19.7%) | 253 (16.7%) | 2583 (17.5%) | |
| Region, n (%) | | | | <0.001 |
| West | 1028 (40.2%) | 1230 (81.4%) | 8435 (57.1%) | |
| Northeast | 352 (13.8%) | 196 (13.0%) | 2814 (19.1%) | |
| South | 890 (34.8%) | 54 (3.6%) | 2463 (16.7%) | |
| Midwest | 285 (11.2%) | 31 (2.1%) | 1061 (7.2%) | |
| Median PSA, ng/mL (IQR) | 5.5 (4.5-6.9) | 5.7 (4.6-7.0) | 5.4 (4.4-6.8) | <0.001 |
| Number of positive cores, n (%) | | | | <0.001 |
| 2 or less positive cores | 1674 (65.6%) | 1044 (69.1%) | 9710 (65.7%) | |
| 3 or more positive cores | 584 (22.9%) | 259 (17.1%) | 3055 (20.7%) | |
| Unknown | 297 (11.6%) | 208 (13.8%) | 2008 (13.6%) | |
| Socioeconomic status, n (%) | | | | <0.001 |
| High SES | 1106 (43.3%) | 1010 (66.8%) | 8983 (60.8%) | |
| Low SES | 1449 (56.7%) | 501 (33.2%) | 5789 (39.2%) | |
| Insurance, n (%) | | | | <0.001 |
| Insured | 2240 (87.7%) | 1313 (86.9%) | 13337 (90.3%) | |
| Medicaid | 143 (5.6%) | 76 (5.0%) | 352 (2.4%) | |
| Uninsured | 59 (2.3%) | 13 (0.9%) | 145 (1.0%) | |
| Unknown | 113 (4.4%) | 109 (7.2%) | 939 (6.4%) | |

IQR= Interquartile range
Percentages may not add up 100% due to rounding.
