# Supplementary material for: Variation in the Use of Active Surveillance for Low-Risk Prostate Cancer Across US Census Regions
Source: Front Oncol. 2021 May 19;11:644885. doi: 10.3389/fonc.2021.644885 (PMC8170083; doi:10.3389/fonc.2021.644885)
Supplement: Supplementary file 2 [file Data_Sheet_2.pdf]

Supplemental Table 2: Multivariable logistic regression analysis assessing receipt of active surveillance between 2010-2016 stratified by age.

		Age<60 (N=21169)	P value	Age 60-70 (N=28949)	P value	Age>=70 (N=10658)	P value
Region*	West	Reference		Reference		Reference	
	Northeast	0.49 (0.45-0.54)	<.0001	0.50 (0.46-0.54)	<.0001	0.53 (0.47-0.60)	<.0001
	South	0.42 (0.38-0.46)	<.0001	0.48 (0.45-0.52)	<.0001	0.77 (0.69-0.87)	<.0001
	Midwest	0.59 (0.52-0.67)	<.0001	0.71 (0.64-0.79)	<.0001	0.93 (0.79-1.10)	0.4022
Age (per one year)		1.03 (1.02-1.04)	<.0001	1.04 (1.03-1.05)	<.0001	1.08 (1.06-1.09)	<.0001
Year							
	2010-2012	Reference		Reference		Reference	
	2013-2015	3.01 (2.79-3.24)	<.0001	2.82 (2.66-2.99)	<.0001	2.41 (2.20-2.63)	<.0001
	2016	5.27 (4.73-5.87)	<.0001	4.44 (4.08-4.83)	<.0001	4.19 (3.64-4.82)	<.0001
Race							
	White	Reference		Reference		Reference	
	Black	1.15 (1.05-1.25)	0.0038	1.25 (1.15-1.35)	<.0001	0.93 (0.81-1.07)	0.3228
	Other/Unknown	1.24 (1.08-1.42)	0.0018	1.10 (0.99-1.22)	0.0834	1.03 (0.87-1.21)	0.7461
PSA (per 1 ng/mL)		0.98 (0.97-1.00)	0.0821	0.98 (0.97-1.00)	0.0349	0.99 (0.97-1.01)	0.3919
Number of positive cores							
	2 or less positive cores	3.45 (3.18-3.74)	<.0001	3.53 (3.31-3.76)	<.0001	3.10 (2.80-3.42)	<.0001
	3 or more positive cores	Reference		Reference		Reference	
	Unknown	1.23 (1.10-1.38)	0.0004	1.37 (1.26-1.49)	<.0001	1.37 (1.21-1.55)	<.0001
Socioeconomic status (Yost index							
	low SES	Reference		Reference		Reference	
	high SES	1.39 (1.29-1.50)	<.0001	1.35 (1.28-1.44)	<.0001	1.52 (1.38-1.67)	<.0001
Insurance							
	Insured	Reference		Reference		Reference	
	Medicaid	0.97 (0.81-1.16)	0.7030	0.85 (0.73-1.00)	0.0512	0.72 (0.56-0.92)	0.0079
	Uninsured	1.43 (1.07-1.91)	0.0144	2.49 (1.91-3.23)	<.0001	1.53 (0.70-3.35)	0.2891
	Unknown	1.13 (0.97-1.33)	0.1229	1.05 (0.94-1.18)	0.3984	0.81 (0.69-0.95)	0.0078
