# Supplementary material for: Variation in the Use of Active Surveillance for Low-Risk Prostate Cancer Across US Census Regions
Source: Front Oncol. 2021 May 19;11:644885. doi: 10.3389/fonc.2021.644885 (PMC8170083; doi:10.3389/fonc.2021.644885)
Supplement: Supplementary file 3 [file Data_Sheet_3.pdf]

Supplemental Table 3: Multivariable logistic regression analysis assessing receipt of active surveillance accounting for the interactions between socioeconomic status and race.

	West	P value	Northeast	P value	South	P value	Midwest	P value
Race and SES interactions								
Race in high SES								
White	Reference		Reference		Reference		Reference	
Black	1.37 (1.20-1.56)	<.0001	0.87 (0.75-1.01)	0.0755	0.97 (0.80-1.18)	0.7755	1.71 (1.20-2.44)	0.0032
Other/Unknown	0.93 (0.84-1.03)	0.1837	1.19 (0.97-1.47)	0.0973	0.99 (0.54-1.83)	0.9759	1.51 (0.70-3.27)	0.2941
Race in low SES								
White	Reference		Reference		Reference		Reference	
Black	1.37 (1.21-1.55)	<.0001	0.51 (0.36-0.73)	0.0003	1.12 (1.00-1.25)	0.0497	1.59 (1.29-1.96)	<.0001
Other/Unknown	1.47 (1.28-1.69)	<.0001	1.17 (0.70-1.93)	0.5505	2.63 (1.62-4.26)	<.0001	1.13 (0.58-2.19)	0.7243
SES								
high vs. low in White	1.54 (1.45-1.63)	<.0001	1.43 (1.21-1.68)	<.0001	1.31 (1.17-1.46)	<.0001	0.83 (0.70-0.98)	0.0297
high vs. low in Black	1.53 (1.30-1.81)	<.0001	2.42 (1.70-3.46)	<.0001	1.14 (0.94-1.37)	0.1894	0.89 (0.61-1.31)	0.5641
high vs. low in Other/Unknown race	0.98 (0.83-1.15)	0.7764	1.47 (0.87-2.47)	0.1501	0.49 (0.23-1.07)	0.0724	1.11 (0.41-3.04)	0.8325
Age	1.04 (1.03-1.04)	<.0001	1.04 (1.03-1.05)	<.0001	1.06 (1.06-1.07)	<.0001	1.06 (1.05-1.07)	<.0001
Year								
2010-2012	Reference		Reference		Reference		Reference	
2013-2015	2.58 (2.44-2.73)	<.0001	3.20 (2.90-3.52)	<.0001	3.06 (2.79-3.35)	<.0001	3.04 (2.61-3.54)	<.0001
2016	3.79 (3.48-4.13)	<.0001	5.88 (5.15-6.71)	<.0001	5.26 (4.63-5.99)	<.0001	6.18 (4.93-7.75)	<.0001
PSA	0.98 (0.97-0.99)	0.0048	1.01 (0.99-1.03)	0.3919	0.97 (0.95-0.99)	0.0108	1.00 (0.96-1.03)	0.8627
Number of positive cores								
3 or more positive cores	Reference		Reference		Reference		Reference	
2 or less positive cores	3.33 (3.14-3.54)	<.0001	3.40 (3.05-3.80)	<.0001	3.65 (3.29-4.05)	<.0001	3.44 (2.90-4.08)	<.0001
Unknown	1.20 (1.11-1.30)	<.0001	1.50 (1.30-1.72)	<.0001	1.67 (1.46-1.91)	<.0001	1.35 (1.04-1.74)	0.0220
Insurance								
Insured	Reference		Reference		Reference		Reference	
Medicaid	0.77 (0.67-0.89)	0.0005	1.25 (0.96-1.63)	0.0964	0.79 (0.61-1.01)	0.0585	1.23 (0.84-1.79)	0.2944
Uninsured	1.16 (0.83-1.62)	0.3896	4.17 (3.01-5.77)	<.0001	1.48 (1.03-2.12)	0.0353	1.29 (0.61-2.72)	0.5003
Unknown	1.77 (1.43-1.95)	<.0001	0.59 (0.52-0.68)	<.0001	1.25 (1.02-1.54)	0.0283	3.17 (2.36-4.24)	<.0001

SES: socioeconomic status
